# PD56 The Cost Implications And Health Outcomes Of Out-Referral Of Drugs On Patients And Public Healthcare Facilities

**DOI:** 10.1017/S0266462325101888

**Published:** 2025-12-29

**Authors:** Raymond Matey

## Abstract

**Introduction:**

Drug out-referral occurs when patients must obtain prescribed medications from external pharmacies due to unavailability or unaffordability at healthcare facility pharmacies. This practice may burden patients financially, lead to health complications, and disrupt the revenue and workflow of healthcare facilities. The study evaluated the economic costs incurred by patients and the potential revenue loss for the University of Ghana Medical Centre (UGMC) due to out-referrals for antihypertensive medications.

**Methods:**

This cross-sectional study took place from September to October 2023. Data on prescribed and dispensed drugs and their prices were collected from the UGMC electronic database and compared with prices from selected local pharmacies. Cost analyses were conducted from both patient and healthcare provider perspectives. Exit interviews with 367 outpatients who experienced drug out-referrals captured financial and productivity cost burden data. Microsoft Excel was used for data analysis, presenting results as frequencies and percentages.

**Results:**

The rate of drug out-referral at UGMC was significant, with 48 percent (5,378/11,220) of prescribed drugs not dispensed at the UGMC pharmacy, a reduction from 54 percent from 2022. Over two months, UGMC’s revenue loss on undispensed antihypertensive drugs alone was GHS43,009.61 (USD3,669.76). Patients referred for antihypertensive drugs incurred a mean direct cost of GHS18.32 (USD1.56) and an indirect cost of GHS26.15 (USD2.23), totaling GHS44.47 (USD3.79) in economic costs, alongside intangible costs such as disappointment (54%), frustration (51%), and stress (54%).

**Conclusions:**

Addressing drug out-referral is essential for enhancing patient care and healthcare facility efficiency. That is, evaluating and highlighting the economic impact of this practice can drive economic growth and innovation to identify cost-effective solutions and improve resource allocation. These insights can strengthen global health systems by ensuring that healthcare practices are both economically viable and beneficial to patient outcomes.

